# A domesticated photoautotrophic microbial community as a biofilm model system for analyzing the influence of plastic surfaces on invertebrate grazers in limnic environments

**DOI:** 10.3389/fmicb.2023.1238913

**Published:** 2023-11-16

**Authors:** Insa Bakenhus, Rense Jongsma, Diana Michler-Kozma, Lea Hölscher, Friederike Gabel, Johannes Holert, Bodo Philipp

**Affiliations:** ^1^Institute for Molecular Microbiology and Biotechnology, Universität Münster, Münster, Germany; ^2^Institute for Landscape Ecology, Universität Münster, Münster, Germany; ^3^Fraunhofer-Institut für Molekulare und Angewandte Ökologie IME, Umweltmikrobiologie, Schmallenberg, Germany

**Keywords:** plastic pollution, photoautotrophic biofilms, grazing, bacteria-microalgae interactions, ecotoxicological test system

## Abstract

The environmental fate of plastic particles in water bodies is influenced by microbial biofilm formation. Invertebrate grazers may be affected when foraging biofilms on plastics compared to biofilms on natural substrata but the mechanistic basis for these effects is unknown. For analyzing these effects in ecotoxicological assays stable and reproducible biofilm communities are required that are related to the environmental site of interest. Here, a defined biofilm community was established and used to perform grazing experiments with a freshwater snail. For this, snippets of different plastic materials were incubated in the photic zone of three different freshwater sites. Amplicon sequencing of biofilms formed on these snippets showed that the site of incubation and not the plastic material dominated the microbial community composition. From these biofilms, individual microbial strains as well as photoautotrophic consortia were isolated; these consortia consisted of heterotrophic bacteria that were apparently nourished by microalga. While biofilms formed by defined dual cultures of a microalga and an Alphaproteobacterium were not accepted by the snail *P. fontinalis*, a photoautotrophic consortium (Co_3) sustained growth and metabolism of this grazer. Amplicon sequencing revealed that consortium Co_3, which could be stably maintained on solid medium under photoautotrophic conditions, reproducibly formed biofilms of a defined composition on three different plastic materials and on glass surfaces. In conclusion, our study shows that the generation of domesticated photoautotrophic microbial communities is a valid novel approach for establishing laboratory ecotoxicological assays with higher environmental relevance than those based on defined microbiota.

## Introduction

Water pollution by plastic is a global issue with largely unknown effects on biota (Dris et al., 2020; Stubbins et al., 2021). When plastic enters aquatic systems it will quickly be colonized by microorganisms. Biofilm formation is, thus, an inevitable process and will influence the further environmental fate of plastic particles in water bodies. Biofilms can influence the sinking of plastic particles to sediments as well as their physiological effects on aquatic animals (Wright et al., 2020; Leiser et al., 2021; Okeke et al., 2022). As biofilms are a substrate for grazing animals in aquatic systems plastic surfaces might impact the grazers’ physiology. An example study for this analyzed the physiology of the snail *Radix balthica* exposed to natural biofilms that had been formed in freshwater (Vosshage et al., 2018; Michler-Kozma et al., 2022). There, the grazing experiments revealed lower biofilm consumption and lower growth rates when the biofilms had been formed on plastic surfaces compared to glass surfaces.

Generally, there are several potential mechanisms by which biofilms formed on plastic may affect invertebrate grazers in a negative way. First, there might be direct toxic effects. Grazing may lead to abrasion of micro- or nanoplastic particles that may be toxic for the forager; additionally, microorganisms in the biofilms can mobilize plastic additives with toxic effects (Fauser et al., 2022; Ockenden et al., 2022). As plastic is known to adsorb chemicals from the water such toxic effects may also arise from exogenous compounds (Yu et al., 2021; Cássio et al., 2022). Additives or adsorbed chemicals may also influence the food quality of biofilms for grazers. Many invertebrate grazers rely on sterols (Shamsuzzama et al., 2020) which are mainly be derived from eukaryotic algae in the photic zone. If microplastic should adsorb alga-inhibiting herbicides the food quality of biofilms might be reduced (Carles et al., 2021; Castro-Castellon et al., 2022). By this mechanism, biofilms on plastic may indirectly contribute to effects of pesticides in aquatic foodwebs (Konschak et al., 2021; Sánchez-Bayo, 2021). The food quality of a biofilm might also be affected if plastic surfaces would be selectively colonized by microorganisms with low food quality. However, to our knowledge, such a selective colonization cannot generally be confirmed far since microbial communities of biofilms on plastic surfaces do not obviously differ from those on natural surfaces (Oberbeckmann et al., 2021). However, low abundancies of plastic-specific OTUs were detected in a large analysis of marine biofilms on plastic (Scales et al., 2021) but this seems to be restricted to the beginning of biofilm formation as plastic surfaces are being masked by biofilms with time. Additionally, nutrient availability may influence the structure of biofilm communities on plastic (Song et al., 2023).

The criteria that apply for ecotoxicological effects for grazers do also apply for microplastic-ingesting biota in a similar way (Alfonso et al., 2023). In this respect, laboratory investigations on ecotoxicological effects of (micro) plastic on aquatic organisms would generally be more meaningful if the plastic is colonized by a biofilm.

Appropriate model systems for ecotoxicological assays should, thus, comprise defined plastic, defined grazers and a defined biofilm. However, while plastic material and grazers can easily be standardized the creation of a defined biofilm is challenging. Standardized laboratory biofilms are mainly designed for testing antimicrobial activity such as antifouling materials and are mainly based on mono-species (Japanese Standards Association, 2010; ASTM International, 2020). *In-situ* biofilm generation can be largely influenced by abiotic conditions; even when these are constant significant variations in biomass content and community structure between replicates cannot be excluded. A reliable determination of key parameters such as C:N ratio or lipid content requires invasive methods that deprive the opportunity of using such biofilms for grazing experiments later.

For reproducible ecotoxicological assays with (micro) plastic synthetic biofilm communities there is the need of defined microorganisms exhibiting reproducible biofilm formation on plastic surfaces. Appropriate microbes for such a defined synthetic biofilm for grazing studies should fulfil certain requirements. First, they should originate from an ecologically relevant habitat. The photic zone would be feasible because many plastic materials are floating and many surface waters which are prone to littering are shallow (ranging, e.g., from puddles via ditches to park ponds). Second, the organisms should be maintainable as mono-cultures on solid media and be able to form biofilms when transferred to liquid medium in which surfaces for biofilm formation are offered. Apart from plastic surfaces they should also form comparable biofilms on a non-plastic reference material such as glass. Third, the envisaged defined synthetic biofilm communities should be stable which could best be achieved if the community members are interdependent. Fourth, the synthetic biofilm community must be accepted by grazers and sustain their growth and reproduction. Considering the sterol auxotrophy of many invertebrates the biofilm communities should contain eukaryotic microorganisms because only few prokaryotes can synthesize sterols (Wei et al., 2016).

Thus, the goal of our study was to obtain microorganisms that show the desired properties under laboratory conditions for establishing a defined community for grazing studies with the freshwater snail *Physa fontinalis*. We focused on limnic systems which have been less explored than marine systems (Latva et al., 2022) but are an important input for marine environments via rivers. We designed a selective strategy to retrieve microorganisms that fulfil the aforementioned requirements. First, *in-situ* enrichments for microorganisms colonizing plastic surfaces were set up in photic zone of water bodies close to dams where flow velocity is low which might enhance colonization (Watkins et al., 2019) and facilitates the installation of devices for biofilm formation. Second, these colonized plastic surfaces were brought to the lab and used as inoculum for selecting microbial communities that colonized a pristine plastic surface under photoautotrophic conditions. The respective microbes forming such a community must be able to detach from a surface and colonize a new one repeatedly. Photoautotrophic condition ensure that microalgae are present that nourish heterotrophic bacteria which is typical for limnic microbial biofilm communities in the photic zone (Gubelit and Grossart, 2020). From these photoautotrophic communities individual strains of microalgae and bacteria should be isolated that can be recombined as communities for producing reproducible biofilms on different plastic materials and glass for grazing studies for addressing the aforementioned goals of this study.

## Materials and methods

### *In situ* incubation setup and sampling

For *in-situ* incubation, research-grade polymer foils made of low-density polyethylene (PE), polyethylene terephthalate (PET) or polystyrene (PS) were used (Goodfellow, Hamburg, DE). Foils were cut into 4 mm × 4 mm × 0.125 mm square snippets using an ethanol-sterilized wire binder (Pavo Sales B.V., Oss, NL) as described previously (Leiser et al., 2021). As containers for the incubation, stainless steel tea strainers (5 cm diameter, Contacto Bander GmbH, Erkrath, DE) were heat-sterilized (200°C, 4 h) and subsequently filled with 100 polymer snippets of a single polymer type. Incubation of the polymer snippets lasted 5 weeks from September 12^th^ to October 17^th^, 2018, at three different sites: *Ems* river (51°57′06.7″ N, 7°59′56.4″ E), Lake *Emssee* (51°57′11.9″ N, 8°00′08.9″ E) and the *Rieselfelder*, an interconnected system of shallow reservoirs which were inundated by a waste water treatment plant effluent (52°01′23.4″ N, 7°39′33.8″ E). The containers were mounted to foamed polystyrene lifting bodies to a depth of about 30 cm in the respective water columns (Supplementary Figure S1). The incubations were weekly sampled by detaching one container per polymer type for measuring chlorophyll fluorescence and biofilm biomass on the snippets (see below). At each sampling site, a sterilized brush was used to remove biomass adhering on the outside of the residual containments for enabling continued sunlight penetration. For transport to the laboratory, the containments were stored at 4°C in heat-sterilized glass beakers. Prior to further processing, snippets were washed three times using sterile phosphate buffered saline (PBS, pH 7.4). After 5 weeks the *in-situ* incubation was stopped and a fraction of the snippets was used for isolating bacteria (see below). To determine the microbial community of the biofilms, additional 15 particles per plastic material and site were pooled and stored at −78°C until further processing for DNA isolation (see below).

### Biofilm-biomass quantification

Biofilm-biomass quantification was performed using crystal-violet staining with protocol modifications adapted from Christensen et al. (1985), Stepanović et al. (2000, 2007) and Arias-Andres et al. (2018). After washing three times with PBS, snippets were transferred to a 24-well microplate with one particle per well. After biofilm fixation at 60°C for 1 h, 0.5 to 1 ml crystal violet (0.3% *w*/*v*) per particle was added (until they were completely submerged) with subsequent incubation on a rocker shaker for 15 min at room temperature. After removal of the crystal violet solution, particles were washed four times with H_2_O_Millipore_. Residual supernatants were removed and 1 ml 33% (*v*/*v*) acetic acid was added. After 20–25 min incubation at 120 rpm, 900 μl of supernatants were transferred into new 24-well microplates for measuring absorption at 595 nm with covered lid using a *Tecan® GENios™ microplate reader* (Tecan Group AG, Männedorf, CH).

### Fluorometric chlorophyll determination

Chlorophyll autofluorescence was measured with the *ChemiDoc™ imager* (Bio Rad Laboratories, Hercules, USA). As excitation light source, *Green Epi Fluorescence* with a wavelength of approximately 550 nm was used (Zecher et al., 2015). Fluorescence emission was detected using the *695/55* filter. Exposure times were manually adjusted to avoid signal overmodulation. Fluorescence intensities were calculated using *Image Lab™* Version 4.1 (Bio-Rad Laboratories) and normalized to area and exposure time.

### DNA extraction and sequencing

For analysing microbial communities analysis of biofilms from *in-situ* incubations and from enriched photoautotrophic consortia, DNA was extracted with the DNA Power Soil Pro Kit (Qiagen, Hilden, DE). Biofilm-covered plastic snippets were transferred into PowerBead Pro Tubes containing 800 μl solution CD1 and shaken horizontally in a vortex adapter for 1 h. After addition of 25 μl proteinase K (22 mg/ml) and incubation at 37°C for 1 h the extraction was continued according to the manufacturer’s instructions. Library preparation, sequencing and data analysis were performed by Microsynth AG (Belgach, Switzerland). Extracted DNA was submitted to two-step PCR amplification of the V4-V5 region of the bacterial 16S rRNA gene, using the primer pair 515F-Y and 926R (Parada et al., 2016). PCR-products were sequenced using a v2 500 cycle kit on the Illumina MiSeq platform. Raw data was submitted to the European Nucleotide Archive (ENA) database and were assigned the Project ID PRJEB45856. For determination of relative abundances of bacterial phyla we performed standard statistical analysis and bioinformatics including the program *R* (R Core Team, 2018).

### Isolation of bacteria from *in-situ* grown biofilms

For isolation of bacteria from *in-situ* grown biofilm, 15 washed snippets of each polymer type were pooled in 2 ml microcentrifuge tubes with 10 sterile glass beads (2.7 mm diameter, Carl Roth GmbH + Co. KG, Karlsruhe, DE). After adding 1 ml of sterile PBS, the tube was vortexed at low speed for 30 s. The resulting supernatant was diluted and plated on solid medium B (Jagmann et al., 2010) supplemented with 0.1% (*v*/*v*) 7-vitamins solution (Pfennig, 1978), 0.01 mM ATP (Bruns et al., 2003) and triple concentrated mixed carbon sources (Cho and Giovannoni, 2004). From these plates, colonies with different morphologies were selected for purifying bacterial strains by repeated transfers on YPG-agar medium [0.075% (*w*/*v*) yeast extract, 0.05% (*w*/*v*) peptone, 0.075% (*w*/*v*) d-glucose, 1.2% (*w*/*v*) Bacto™ Agar (Becton Dickinson GmbH, Heidelberg, DE); adapted from Bruns et al. (2003)]. All cultivation steps were performed at room temperature.

### Enrichment of algal-bacterial consortia and isolation of microorganisms therefrom

For enrichment and cultivation of photoautotrophic algal-bacterial consortia from *in-situ* grown PE biofilms, modified Diatom Medium (DM; pH 6.75/HCl; adapted from Cohn and Pickett-Heaps, 1988 and Cohn et al., 2003) was used in which soil extract, FeSO_4_ and MnCl_2_ was replaced by 0.1% (*v*/*v*) f/2 trace element solution (Guillard and Ryther, 1962). After autoclaving, 0.1 mM NaHCO_3_ and DM vitamin solution were added to the medium. For enrichment cultures, 15 washed PE snippets were pooled in a 2 ml microcentrifuge tube with 10 sterile glass beads (see above) in 1 ml of sterile PBS and vortexed at low speed for 30 s (Supplementary Figure S2). After removal of the supernatant, the snippets were washed three times in PBS. Single snippets were then used to inoculate 1 ml of DM in a 24-well microplate containing a sterile pristine PE snippet. These microtiter plates were incubated for 12 days at 21°C with 180 rpm in a light incubator (Phytobiochamber Model EGCS 701, EQUiTEC; light source: Lumilux Warmwhite, Osram, DE; photon flux density: approx. 90 μE m^−2^ s^−1^; light/dark cycle: 14: 10 h). For transferring the enrichment cultures, 20 μl of the suspended fraction were used to inoculate 980 μl DM containing two pristine PE snippets in a microtiter plate. After 12 days of incubation, when the pristine PE snippets were covered with a chlorophyll-containing biofilm, this transfer procedure was repeated. After a total of three transfers, six biofilm-covered polymer particles were streaked onto solid DM and cultivated in the light incubator. The resulting photoautotrophic algal-bacterial consortia were re-plated onto new solid DM every 1 to 2 weeks.

For isolation of heterotrophic bacteria from algal-bacterial consortia, cell material from the consortia was streaked onto solid medium B supplemented with 2 mM of each d-glucose, *N*-acetyl-d-glucosamine and sodium glycolate (MB3G). Plates were incubated in the dark. For isolation of photoautotrophic microalgae from consortia, cell material was streaked onto solid DM and transferred with weekly changing antibiotics [in chronological order: 260 μg/ml disodium carbenicillin and 60 μg/ml monosodium ampicillin, 50 μg/ml streptomycin sulfate, 10 μg/ml gentamycin sulfate, 50 μg/ml kanamycin sulfate; adapted from Cottrell and Suttle (1993)]. Plates were incubated in the light as described above for the enrichment cultures. Axenity of the algal isolates was tested by 4′,6-diamidino-2-phenylindole (DAPI) staining and by cultivation on MB3G in the dark. The resulting axenic algal isolates were re-plated onto new solid DM with 260 μg/ml disodium carbenicillin and 60 μg/ml monosodium ampicillin every 2 to 4 weeks for maintenance.

### Identification of microorganisms

For taxonomical classification of isolated bacterial strains, DNA was isolated with the *Gentra Puregene Yeast/Bact. Kit* (Qiagen, Hilden, DE) according to the manufacturer’s instructions. Purified genomic DNA was amplified using the primer pair *16S_27_fw* (5’-AGAGTTTGATCATGGCTCA-3′) and *16S_1492_rev* (5’-TACGGTTACCTTGTTACGACTT-3′, adapted from Weisburg et al., 1991). PCR-amplified DNA was purified with the *GeneJET PCR Purification Kit* (Thermo Fisher Scientific, Waltham, USA) and sequenced by Eurofins Genomics (Ebersberg, DE) with the *Mix2Seq Kit*.

For taxonomical classification of isolated algal strains, DNA isolation was performed according to the protocol described by Jagielski et al. (2017). For cell lysis, glass beads (d: 400–600 μm; Sigma Aldrich, St. Louis, USA), Mikro-Dismembrator S (Sartorius AG, Göttingen, DE), 300 μl of 5 M NaCl and 240 μl CTAB buffer were used. After adding the phenol/chloroform/isoamylalcohol solution, additional 10 s vortexing and subsequent centrifuging at 16,699 × *g* for 1 min were implemented.

After DNA precipitation, centrifugation was performed with 18,407 × *g.* Purified DNA was PCR-amplified using the primer pairs CHLORO_fw (5′-TGGCCTATCTTGTTGGTCTGC-3′)/CHLORO_rev (5′-GAATCAACCTGACAAGGCAAC-3′; Gumbi et al., 2017), ITS1_fw (5′-AGGAGAAGTCGTAACAAGGT-3′)/ITS4_rev (5′-TCCTCCGCTTATTGATATGC-3′; Hadi et al., 2016), rbcL_192_fw (5′-GGTACTTGGACAACWGTWTGGAC-3′)/rbcL_657_rev (5′-GAAACGGTCTCKCCARCGCAT-3′; Hadi et al., 2016), rbcL_375_fw (5′-TTTGGTTTCAAAGCIYTWCGTGC-3′)/rbcL_1089_rev (5′-ATACCACGRCTACGRTCTTT-3′; Hadi et al., 2016), tufA_fw (5′-TGAAACAGAAMAWCGTCATTATGC-3′)/tufA_rev (5′-CCTTCNCGAATMGCRAAWCGC-3′; Hall et al., 2010), tufA_50_fw (5′-TGGATGGTGCTATTYTAGTTG-3′)/tufA_870_rv (5′-ATAGTGTCRCCTGGCATAGC-3′; Hall et al., 2010). PCR-amplified DNA was purified and sequenced as described above.

For phylogenetic affiliation analysis, the BLASTn suite search tool (Altschul et al., 1990) was used.

### Growth experiments with photoautotrophic consortia

Growth experiments with photoautotrophic consortia were performed for re-colonization experiments and for grazing experiments. In both cases, pre-cultures were set up by resuspending cell material from consortia growing on solid DM in liquid DM and incubated for 7 days.

For the re-colonization experiments, the photoautotrophic consortia Co_1 to Co_6 were used. Pre-cultures were inoculated from agar plates in 20 ml DM in 100 ml Erlenmeyer flasks at 175 rpm and 21°C in the light incubator (EQUiTEC). For main cultures, 800 μl DM were inoculated with 200 μl pre-culture in 24-well plates (*Thermo Scientific™ Nunc™ Cell-Culture Treated Multidishes* [Thermo Fisher Scientific Inc., Waltham, USA]) containing PE, PET or PS snippets, which had prior been sterilized by bathing in in ethanol (70% v/v) for 15 min followed by drying and 5 min UV-irradiation in a laminar-flow sterile bench before use. After 7 days incubation, chlorophyll fluorescence and biofilm biomass on the snippets were determined as described above.

### Cultivation of consortium Co_3 for grazing experiments

For grazing experiments, consortium, Co_3 was used. Pre-cultures were grown in 125 ml DM in 500 ml Erlenmeyer flasks without shaking at room temperature and daylight. As biofilm surfaces, slides of 2.5 cm × 8 cm were cut from PE-, PET- and PS foils (Goodfellow, thickness: 0.125 mm), which were roughened on one side using 60 grid sandpaper. Before use, polymer slides were sterilized in 70% (*v*/*v*) ethanol for 30 min, washed in sterile H_2_O_demin_ and stored in sterile H_2_O_demin_ at 4°C. As control material, fully frosted glass microscope slides were used which had prior been rinsed with H_2_O_demin_ and autoclaved. Before inoculation of main cultures, pre-cultures were washed via centrifugation of 50 ml at 6,000 rpm for 30 min. Supernatants were carefully discarded, and pellets were resuspended in sterile DM-medium to a volume with a chlorophyll concentration of 1 μg/ml using the corresponding chlorophyll fluorescence intensities determined for the diatom *Phaeodactylum tricornutum* as described by Zecher et al. (2015).

For the main cultures, in which biofilm formation should occur, four square petri dishes per material type were filled with 50 ml of the washed cell suspension. In each petri dish, four polymer or glass slides were placed with the rough side facing upwards. The petri dishes were incubated for 7 days at room temperature and daylight. Biofilm formation was evaluated via chlorophyll fluorescence as described above. For analysing shifts in the bacterial community compositions via amplicon sequencing, cell material was isolated from Co_3 on solid DM media from three transfers (January 2020, August 2020, and March 2021), 7-day-old biofilms on different plastic types and from the respective surrounding supernatants before grazing started. DNA-extraction and 16S rDNA amplicon sequencing were performed as described above. To confirm that the isolated algae Alg_3.1 was originally present in Co_3 its 18S rRNA sequence was aligned against the R2 mate samples of the amplicon sequencing data of Co_3 from the three transfers using bowtie2 using the “sensitive-local” algorithm (version 2.5.1, https://github.com/BenLangmead/bowtie2).

### Grazing experiments

Plastic and glass slides with biofilms of Co_3, which were produced in the growth experiments described above, were placed in 1,000 ml glass beakers with 500 ml synthetically reconstituted surface freshwater (Osterauer et al., 2010) and 4 individuals of the gastropod *Physa fontinalis*. Snails obtained from a commercial distributor for aquarist supplies, who kept the snails in quarantine had a length of 3.8 ± 0.5 mm (mean ± standard deviation). The slides were replaced every 3 ½ days with slides from the same batch stored at 4°C in the dark. Experiments lasted for 3 weeks in a climate-controlled room at 20°C with a 16:8 h cycle. The physiological parameters of *P. fontinalis* were determined in weekly intervals, including: size measured by a digital caliper as well as a digital microscope (VHX-5000, Keyence Corp.), mortality, faeces dry mass and the numbers of eggs and egg packages. Biofilms were analysed by measuring chlorophyll fluorescence before and after the grazing experiments.

## Results

### Composition of *in-situ* biofilm communities is dominated by the incubation site and not by the plastic material

**Figure 1:**
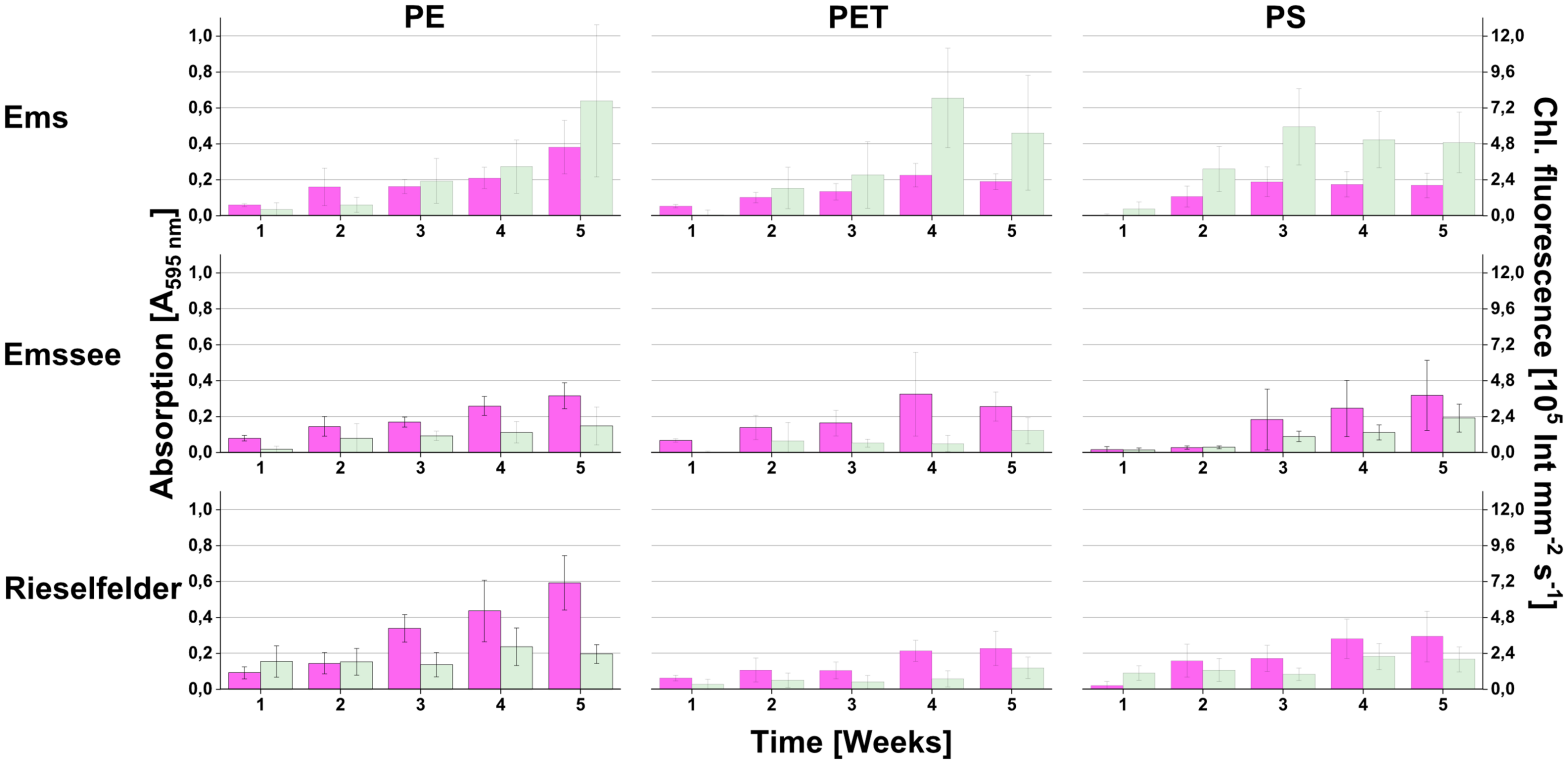
Colonization of plastic snippets during the in the *in-situ*-incubation at three different freshwater sites (*Ems*, *Emssee*, and *Rieselfelder*). At each sampling event, individual snippets were first used for measuring chlorophyll fluorescence (green bars) before they were used for measuring biofilm biomass with crystal violet (magenta bars). Error bars indicate standard deviation (*n* = 12).

The microplastic snippets of all three materials (PE, PET, PS) were colonized with chlorophyll-containing biofilms over time on all three incubation sites (Figure 1). The chlorophyll intensities were generally lower with the *Emssee* and *Rieselfelder* samples compared to the *Ems* samples. Microscopic examination revealed the presence of microalgae (various diatoms and *Chlorella*- and *Chlamydomonas*-like morphotypes) and of prokaryotic cells (not shown).

From all three incubation sites, strains of heterotrophic bacteria could be isolated by direct plating of sheared biofilm material (Supplementary Table S1). Most isolated strains belonged to the Alpha-, Beta- and Gammaproteobacteria including genera of characteristic freshwater bacteria such as *Gemmobacter*, *Mitsuaria*, and *Aeromonas*, respectively. Typical freshwater Bacteroidetes, such as *Flavobacterium* spp., could also be isolated.

**Figure 2:**
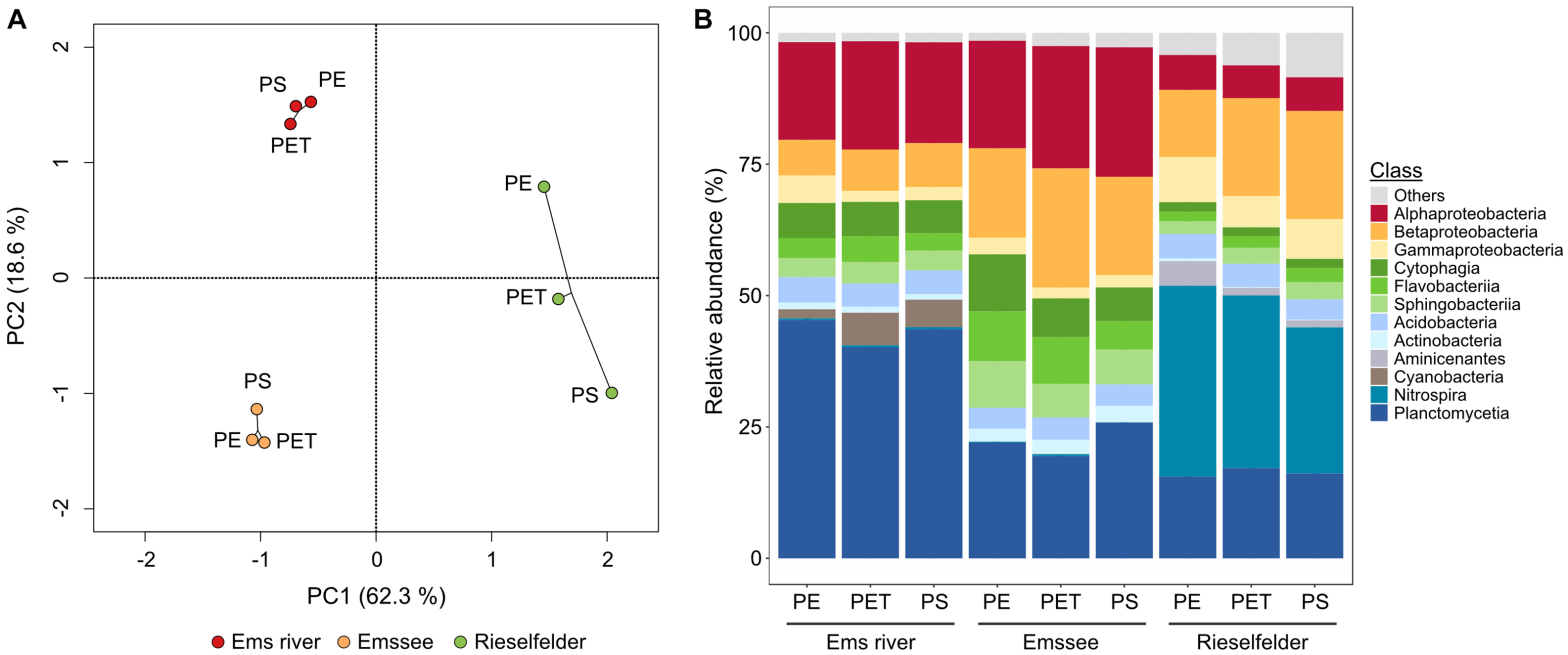
Analysis of biofilm communities on plastic snippets from *in-situ* incubations via amplicon-sequencing of genes for the 16S rRNA on the class level. **(A)** Statistical evaluation via principle-component analysis (PCA); **(B)** Relative abundances. The analysis was performed with 15 plastic snippets per polymer and location after 5 weeks of incubation at the indicated locations. Classes with <1% abundance in all samples are summarized as “others”.

The cultivation-independent analysis of the biofilm communities exhibited a much greater diversity than the cultivation analysis (Figure 2). While Alpha-, Beta- and Gammaproteobacteria and Bacteroidetes were also found to be abundant, the amplicon sequencing revealed a large proportion of *Planctomycetia* that were not retrieved by cultivation (Figure 2B). Furthermore, the *Rieselfelder* samples contained a large percentage of members of the genus *Nitrospira*, which were not detected in the biofilms from the other incubation sites. Statistical analysis of the cultivation-independent analysis showed that the bacterial communities clustered according to the incubation sites rather than the plastic material, on which the biofilm had formed (Figure 2A).

### Selection for biofilm-forming photosynthetic communities results in stable photoautotrophic consortia maintainable on solid media

For isolating microorganisms from these *in-situ* enrichments that could be used for producing defined biofilms for grazing experiments we set up laboratory enrichments under photoautotrophic conditions in the next step.

**Figure 3:**
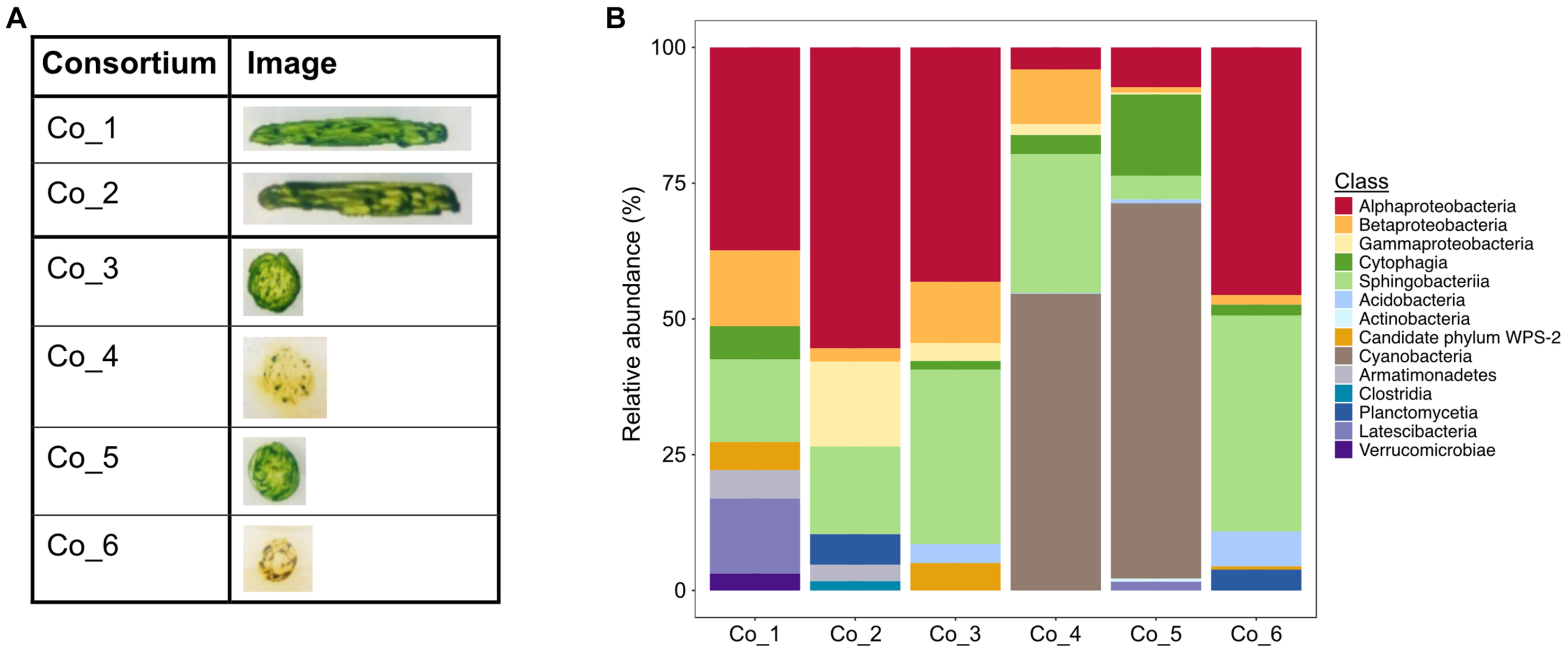
Photoautotrophic consortia enriched from *in-situ* incubations. **(A)** Macroscopic photographs of consortia on solid medium; **(B)** Analysis of consortia via amplicon-sequencing of genes for the 16S rRNA on the class level.

These laboratory enrichments were inoculated with plastic snippets from which loosely attached microorganisms had been detached by gentle shearing forces (Supplementary Figure S2). In the following, we selected for microorganisms that were able to colonize pristine plastic snippets under photoautotrophic conditions. We restricted the enrichment to PE snippets because biofilms on this material showed the highest biofilm-biomass in the *in-situ* incubations (Figure 1). This procedure led to the enrichment of communities that reproducibly re-colonized PE-surfaces. After 3 transfers, plastic snippets with biofilms were used to inoculate solid media. By this procedure, we obtained photoautotrophic microbial communities that could be maintained on agar plates by regular transfer about every fortnight since November 2018. In total, we enriched six consortia: two originating from *Rieselfelder* (Co_1 and Co_2) and four from *Ems* river PE-particles (Co_3 to Co_6; Figure 3A). In comparison to *in-situ* grown biofilms the consortia had originated from, the bacterial communities within the consortia displayed a smaller diversity (Figure 3B). The phylum of Planctomycetes, which exhibited large proportions in the *in-situ* grown biofilms (Figure 2B) was only represented in Co_6 and Co_2 at low abundances. The phylum of *Nitrospira* was not present in the algal-bacterial consortia derived from the *Rieselfelder* while it was abundant in the *in-situ* sample. Cyanobacteria, represented in comparably small abundances within *in-situ* grown biofilms in the *Ems* river, dominated the algal-bacterial consortia Co_4 and Co_5, derived from PE biofilms grown in the *Ems* river. In Co_3 and Co_1, we also observed the yet uncultivated candidate phylum WPS-2. The presence of microalgae in the consortia was verified by microscopy (not shown).

### Consortium Co_3 efficiently re-colonizes plastic surfaces

**Figure 4:**
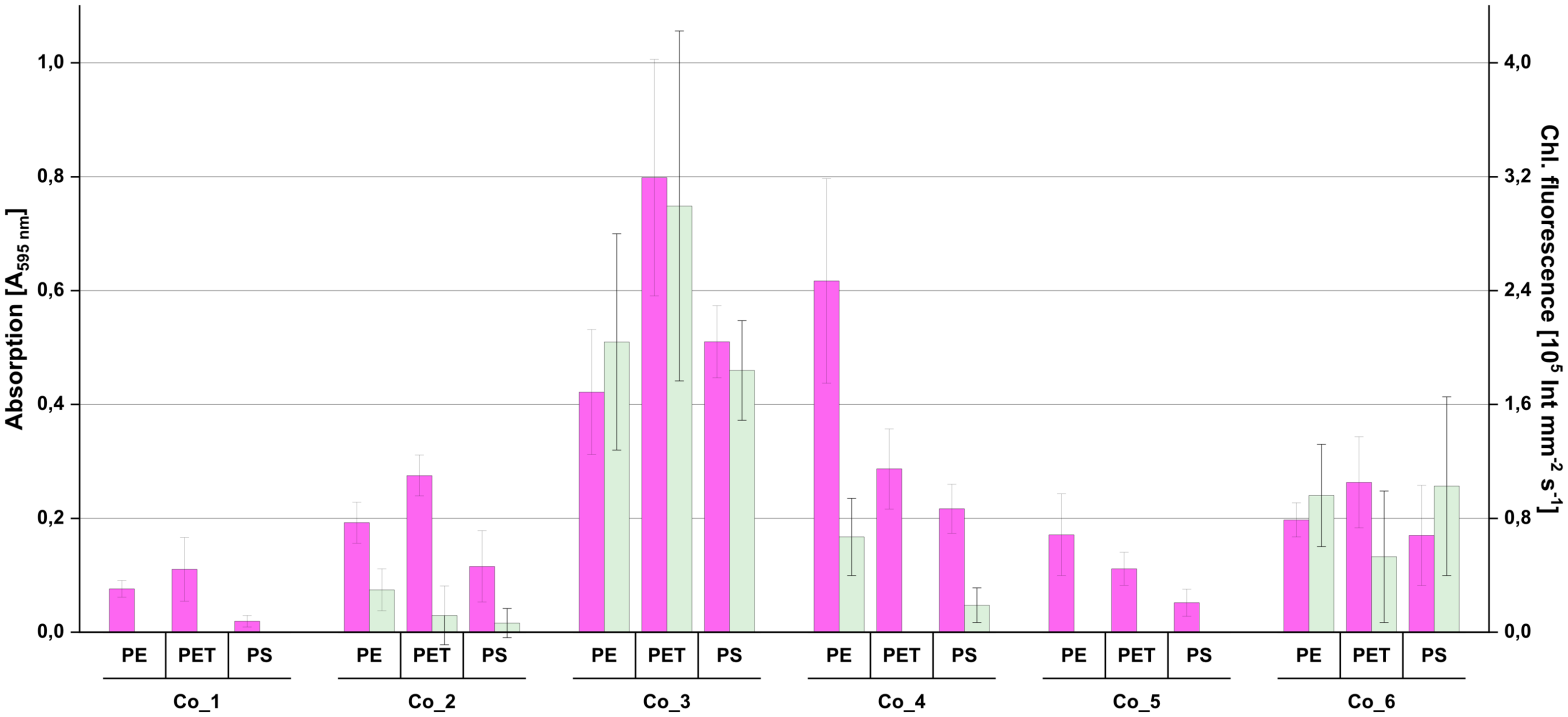
Re-colonization of plastic surfaces by the individual photoautotrophic consortia shown in Figure 3. Individual plastic surfaces were first used for measuring chlorophyll fluorescence (green bars) before they were used for measuring biofilm biomass with crystal violet (magenta bars). Error bars indicate standard deviation (*n* = 8).

For investigating whether the consortia were able to re-colonize plastic surfaces from liquid culture after being maintained on agar plates and for identifying the consortium with the highest biofilm production, re-colonization experiments were performed with all three plastic polymers (PE, PET and PS) originally used in the *in situ* incubation (Figure 4). The consortia showed large differences in biofilm formation when exposed to plastic surfaces. Co_3, which was derived from a biofilm on PE snippets from the *Ems* river, showed the highest chlorophyll fluorescence values and highest biofilm biomass. While all consortia showed at least some biofilm biomass, there was no chlorophyll fluorescence detected in Co_1 and 5, and also only low values in other consortia.

**Figure 5:**
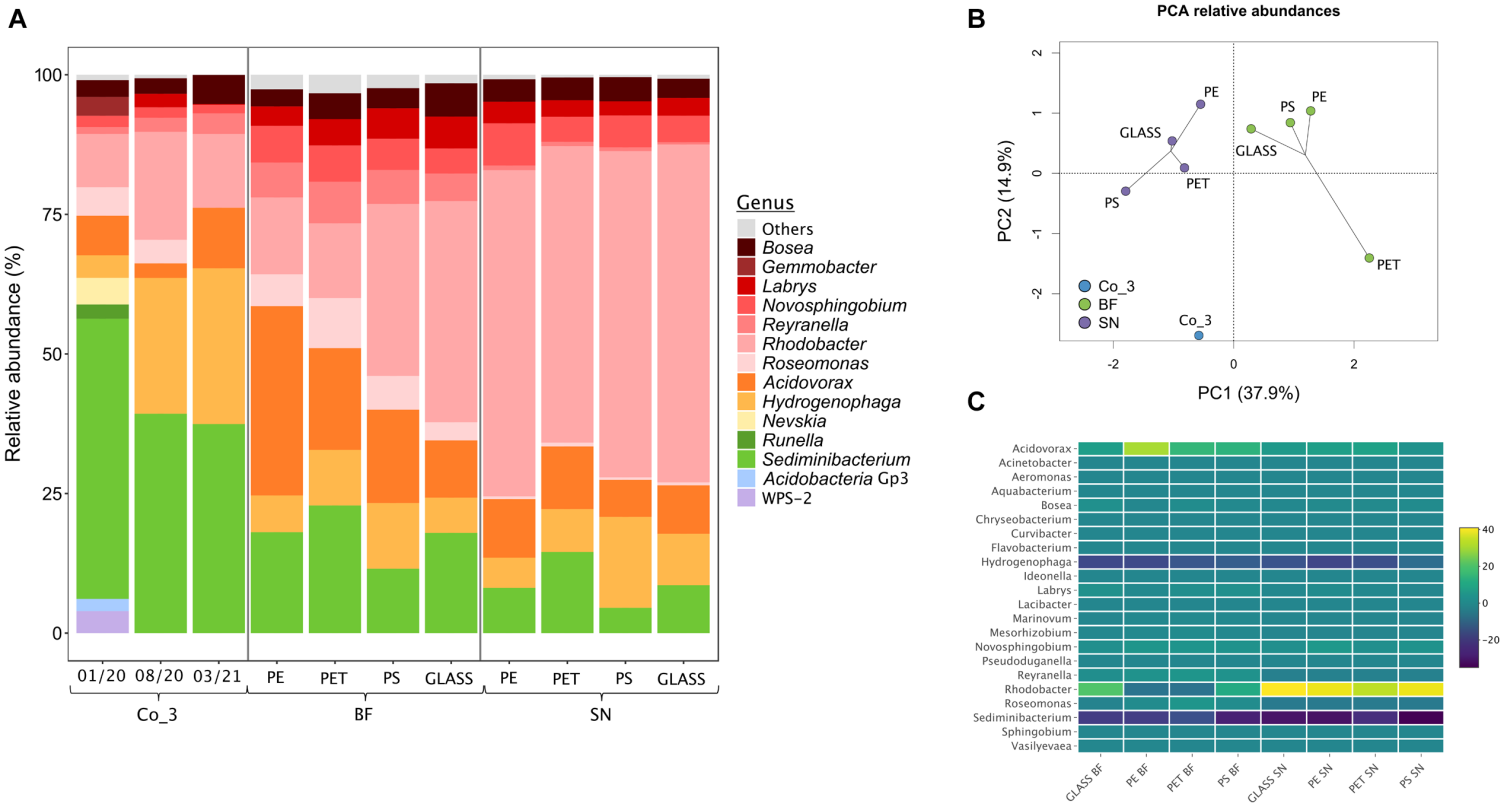
Analysis of the photoautotrophic consortium Co_3 via amplicon-sequencing of genes for the 16S rRNA on the genus level. **(A)** From left to right: relative abundancies of genera in Co_3 after maintenance on solid diatom medium for 2, 10 and 16 months after initial isolation; abundancies during a growth experiment for providing biofilms for a grazing experiment: the community composition of the consortium on solid diatom medium directly before inoculating the growth experiment (Co_3 08/20) was compared to the composition of the biofilm fraction (BF) and of the supernatant (SN) for each material. Genera with <2% abundance in all samples are summarized as “others”; **(B)** Statistical evaluation via principle-component analysis (PCA); **(C)** Heat map displaying the relative abundances within biofilm and the supernatant relative to the consortium Co_3 (08/20) on solid medium on the genus level. For each material, 3 replicates were used from which the extracted DNA was pooled.

For further characterizing the composition and stability of Co_3, we repeatedly submitted it to amplicon sequencing. These analyses showed a reduction of the diversity on class and genus levels with time. The candidate genus WPS-2 disappeared within about 8 months while members of genera with cultivated representatives remained (Figure 5A).

### Biofilms formed by a defined dual-species culture are not accepted by the grazer

As Co_3 should contain microorganisms capable of re-colonizing a plastic surface from a culture with suspended cells, we isolated algal and bacterial strains from this consortium. For this, cell material from the consortia plates were transferred to solid media and growth conditions that favor the growth of axenic microalgae and heterotrophic bacteria, respectively. The procedure for obtaining axenic microalgae led to the isolation of strain Alg_3.1, which was classified as *Chlamydomonas* sp. Alignment of the 18S rRNA of the isolate to the amplicon sequencing data from Co_3 confirmed that Alg_3.1 was present in the original Co_3 consortium and in the transfers on solid DM medium (alignment rates of 4.40, 2.78, and 6.42% in January 2020, August 2020, and March 2021, respectively). The procedure for obtaining heterotrophic bacteria, which was also applied to the other consortia, led to the isolation of several strains of Alphaproteobacteria of the genus *Gemmobacter* and Betaproteobacteria of the genus *Acidovorax* (Supplementary Table S2). Apart from further proteobacterial strains, a member of the genus *Flectobacillus* (Bacteroidetes) was isolated. Pre-test with a number of these bacterial strains in co-culture with *Chlamydomonas* sp. strain Alg_3.1 revealed biofilm formation of several isolates in co-culture with *Chlamydomonas* sp. Alg_3.1 on PET (not shown). For further colonization experiments, *Gemmobacter* sp. strain O was then chosen because it showed reliable biofilm formation on different plastic surfaces. Biofilm formation and algal growth could be largely reduced when co-cultures were supplied with succinate as growth substrate for the bacterium indicating that biofilm formation was optimal when the co-culture relied on photoautotrophic conditions (Supplementary Figure S3). However, these dual-species biofilms were not grazed by our model invertebrate *Physa fontinalis* (not shown).

### Consortium Co_3 forms reproducibly biofilms on different materials that are grazed by *P. fontinalis*

Due to the stability of the community composition together with the recolonization capacity biofilms of Co_3 were tested as a substrate for the grazer *P. fontinalis*. For this, we set up biofilm experiments with Co_3 by offering three types of plastic (PE, PET, PS) and glass as surfaces as control. These experiments showed that Co_3 reproducibly formed biofilms for the test period of 8 weeks (Supplementary Figure S4). During this time, *P. fontinalis* showed growth and activity for a period of 3 weeks (Table 1). Snails grazed on the biofilms leading to small but continuous growth rates, reproduction and faeces excretion. There were no significant differences in snail performance among the different substrates.

**Table 1:** Performance of the freshwater snail *Physa fontinalis* during grazing on biofilms formed by the photoautotrophic consortium Co_3 on different surfaces.

Parameter	Glass (m ± stdev)	PE (m ± stdev)	PET (m ± stdev)	PS (m ± stdev)
Snail growth	0.02 ± 0.03	0.03 ± 0.06	0.05 ± 0.05	0.05 ± 0.07
Reproduction	2.13 ± 1.36	1.63 ± 1.60	1.25 ± 1.16	2 ± 2.62
Faeces mass	4.64 ± 1.17	2.56 ± 1.42	1.74 ± 0.27	1.89 ± 0.36

Growth rates (mm per snail per week), reproduction (number of eggs per snail per week) and faeces mass (mg per snail per week) were followed for 3 weeks. +/− indicates standard deviation (*n* = 96).

For further assessing the suitability of using Co_3 as model community we analyzed the community composition directly before the snails were added. Statistical analysis of the communities within the biofilm and the suspended fraction by amplicon sequencing revealed that the composition clustered according to the individual suspended and biofilm fractions rather than according to surface material (Figures 5A,B) as was also previously observed in the *in-situ* incubations. A heat-map analysis of the community structure indicated that the largest difference to the stock consortium of Co_3 on solid medium was the strong reduction of *Sediminibacterium* sp. and *Hydrogenophaga* sp.in the biofilm and the suspended fraction as well as a strong increase of *Rhodobacter* sp. in all suspended fractions (Figure 5C).

## Discussion

Studies on the ecotoxicity of (micro) plastics on aquatic organisms should consider the influence of biofilms. However, biofilms are difficult to standardize, and there is demand on obtaining reproducible biofilm systems that have ecological relevance. In this study, we established a stable photoautotrophic microbial consortium that reproducibly formed biofilms on different surfaces which could be used as food for the grazing snail *Physa fontinalis*. This consortium Co_3 originated from the photic zone of the *Ems* river and consisted of a *Chlamydomona*s strain and about 20 species of heterotrophic bacteria of which members of the genera *Roseomonas* (Alphaproteobacteria), *Hydrogenophaga* (Betaproteobacteria) and *Sediminibactrium* (Chitinophagaceae) were dominating community members.

Initially, we aimed at establishing a defined co-culture that can be recombined on demand from mono-cultures of photoautotrophic microalgae and heterotrophic bacteria isolated from biofilms on plastic from the photic zones of dams and reservoirs. Such defined dual cultures of individually maintainable strains that formed reliably biofilms could indeed be established but were, unfortunately, not accepted by the grazer. Based on the unexpected finding that enriched photoautotrophic consortia could be stably propagated on solid media we obtained the consortium Co_3 that showed reproducible biofilm formation on different plastic materials and could also serve as food for the grazing snail *P. fontinalis*. Importantly, Co_3 also formed biofilms on glass which offers an opportunity to analyze plastic-specific effects of this model biofilm (Wright et al., 2020).

Functional and genetic analyses of consortium Co_3 during a grazing experiment showed that it could effectively nourish *P. fontinalis* while its community structure changed only moderately in the quantity but not in the quality of its individual members. Thus, Co_3 showed a stable phenotype and genotype irrespective of the localization of cells (planktonic or in the biofilm) and of the surface material. Based on this stability and considering a broader biological definition of domestication as (Purugganan, 2022) we propose the term domesticated community for our biofilm model community.

The community structures of the *in-situ* enrichments were apparently not influenced by the plastic material but rather by the conditions at the incubation site which is also observed with biofilms on plastic in marine sites (Oberbeckmann et al., 2021). In this respect, the large proportion of *Nitrospira* in the biofilms from the Rieselfelder site, which was not observed for the other incubation sites, might have been influenced by the outflow of a large municipal sewage treatment plant in *Münster-Coerde* nearby. In the six consortia obtained by the subsequent laboratory enrichment growth of the heterotrophic bacteria relied on cross-feeding of exudates from the photoautotrophic microalgae. However, analysis of the six consortia showed that our selection for photoautotrophic growth and surface adhesion can have different outcomes regarding the structure of the communities and their ability to colonize plastics. The follow-up assay for re-colonization of surfaces from the planktonic phase after being inoculated from solid medium was therefore crucial for identifying a community with the desired prerequisites outlined in the introduction.

Within continued cultivation on solid media, the diversity of Co_3 was reduced which might have a variety of reasons ranging from adverse abiotic conditions (higher temperatures etc.) to interruption of nutrient transfer (Overmann et al., 2017). Remarkably, however, bacteria of the so-far uncultured candidate phylum WPS-2 could be propagated in the first cultures of Co_3 on solid media indicating that the domestication of such photoautotrophic microbial communities might be a way of increasing the cultivability of environmental bacteria. As bacteria of the candidate phylum WPS-2 are believed to use organic substrates that may also be exudated by algae (Sheremet et al., 2020) they might have been out-competed by other taxa which used the same substrates.

Compared to Co_3 on agar plates, the abundance of *Sediminibacterium* sp. and of *Hydrogenophaga* sp. decreased in both the biofilm and the suspended fractions in the grazing experiment while the abundance of *Rhodobacter* sp. increased (Figures 5A,C). This could indicate that *Sediminibacterium* sp. and *Hydrogenophaga* sp. were more important when the community is growing at a moist surface-liquid interface compared to the submerged situation where *Rhodobacter* sp. might have important functions. In agreement with preferred growth in biofilms, a *Sediminibacterium* strain showed upregulation of stress-related proteins in planktonic cells compared to aggregated cells (Ayarza et al., 2015).

Besides the obvious cross feeding of exudates interactions between photoautotrophic microalgae and heterotrophic bacteria can be very diverse as evidenced from natural communities (Amin et al., 2012) as well as synthetic communities for both marine (Deng et al., 2022) and limnic conditions (Lamprecht et al., 2022). We do not know the basis for the stability of Co_3 but the stable maintenance of the bacterial members suggests that bacteria are likely to support growth of the algae in a specific way.

In many natural assemblages of this type, heterotrophic bacteria feed vitamin B12 to the algae (Amin et al., 2012) but this was most probably not essential because the *Chlamydomonas* sp. from Co_3 grew also axenically without bacteria. Other metabolic interactions by which algae-associated bacteria may support growth of their photoautotrophic community member could be the degradation of organic nitrogen compounds such as methylamines and glycine betaine to ammonium that can be used by the algae. This property is apparently frequent among marine algae-associated members of the *Rhodobacteraceae* family (Zecher et al., 2020). In this respect, the repeated isolation of *Gemmobacter* spp. which is described as a common freshwater microalgae-associated bacterium of this family (Zhang et al., 2020) and is also capable of methylamine-utilization (Kröber et al., 2021) might suggest ammonium cross-feeding from bacteria to the algae is involved in our communities as well. Interestingly, Co_3 grew with monomethylamine as sole nitrogen source in liquid culture (not shown). Furthermore, metabolic interactions between microalgae and their associated bacteria might stimulate biofilm formation itself as it was also observed in other cases and can rely on a multitude of factors (Grossart et al., 2006; Windler et al., 2015).

In our study with Co_3 and *P. fontinalis*, we did not observe an influence on the fitness of the snails indicating under these conditions no harmful additives were mobilized or leached from the plastic material. This might partly be due to the fact that we did not use weathered plastics from which additives and adsorbed chemicals might leach more easily (Rummel et al., 2019; Luo et al., 2023).

As a main conclusion, our results show that domesticated communities might be a very promising approach for standardizing mixed-species biofilms for ecologically relevant ecotoxicological assays. It is compellingly easy for obtaining and maintaining a model community, at least for a defined series of experiments. While our domesticated community was genetically and phenotypically stable in the described timeframe (and it still is) it would certainly be even better to have a completely defined consortium that can be synthetically rearranged from individual stock cultures on demand, especially for exchange between labs and for certifiable protocols. In this respect, we tried to isolate the main representatives of Co_3 but failed to do so thus far (not shown). This may indicate that metabolic interdependencies of the community members are relatively strong and that the underlying nutritional requirements are difficult to reconstitute in mono-cultures.

## Data availability statement

The datasets presented in this study can be found in online repositories. The names of the repository/repositories and accession number (s) can be found at: https://www.ebi.ac.uk/ena, PRJEB45856.

## Author contributions

IB conceptualized, performed, and evaluated the amplicon sequencing and contributed to the writing of manuscript together with RJ and BP. RJ conceptualized, performed, and evaluated all cultivation-based experiments and supported amplicon sequencing. Contributions of IB and RJ were equal. DM-K performed the grazing experiments. LH performed the isolation of algae and the re-colonization experiment. FG conceptualized and managed the project and supported writing the of manuscript. JH supported the evaluation of amplicon sequencing and writing of the manuscript. BP conceptualized and managed the project and wrote the manuscript. All authors contributed to the article and approved the submitted version.
