# Supplementary material for: A domesticated photoautotrophic microbial community as a biofilm model system for analyzing the influence of plastic surfaces on invertebrate grazers in limnic environments
Source: Front Microbiol. 2023 Nov 16;14:1238913. doi: 10.3389/fmicb.2023.1238913 (PMC10687189; doi:10.3389/fmicb.2023.1238913)
Supplement: Supplementary file 1 [file Data_Sheet_1.pdf]

Supplementary Material for

A domesticated photoautotrophic microbial community as a biofilm model system for analyzing the influence of plastic surfaces on invertebrate grazers in limnic environments

Insa Bakenhus[1+], Rense Jongsma[1+], Diana Michler-Kozma[2], Lea Hölscher[1], Friederike Gabel[2], Johannes Holert[1], Bodo Philipp[1,3]

[1]Westfälische Wilhelms-Universität Münster, Institute for Molecular Microbiology and Biotechnology, Münster, Germany

[2]Westfälische Wilhelms-Universität Münster, Institute for Landscape Ecology, Münster, Germany

[3]Fraunhofer-Institut für Molekulare und Angewandte Ökologie IME, Schmallenberg, Germany

*Corresponding author: Bodo Philipp

E-Mail: bodo.philipp@uni-muenster.de; Tel.: (+49) 251 8339827; Fax: (+49) 251 8338388; Adress: Institut für Molekulare Mikrobiologie und Biotechnologie, Westfälische Wilhelms-Universität Münster, Corrensstr. 3, 48149 Münster, Germany.

[+]These authors contributed equally to the study.

Supplementary material

Materials and methods: Growth experiments with bacterial and algal co- and mono-cultures

Growth experiments with bacteria and *Chlamydomonas* sp. strain Alg_3.1 were performed with 1 mL DM in 24-well microplates (*Standard TC* [Sarstedt AG & Co. KG, Nümbrecht, DE); cultures were incubated with sterilized 10 mm^2 PET, PE and PS snippets (Online-Plast, Koblenz, DE] at 180 rpm and 21 °C in a light incubator (EQUiTEC). Algal and bacterial pre-cultures were inoculated from solid media. Algal pre-cultures were incubated for 7 days and contained with 260 µg/mL carbenicillin and 60 µg/mL ampicillin in DM; to remove the antibiotics before inoculating main cultures, algal pre-cultures were washed by centrifugation with 3000 × *g* for 10 min and resuspension in fresh DM after decanting the supernatant. Bacterial pre-cultures were grown overnight (12-14 h) with a 4 mL of a complex medium containing 0.15% yeast extract, 0.1% peptone and 0.15% glucose at 30°C with shaking at 200 rpm. For main mono- or co-cultures with alga, 0.8 mL DM was inoculated with 0.2 mL of the washed pre-culture. For main mono- and co-cultures with bacteria, pre-cultures were washed by centrifugation at 7300 × *g* for 5 min and resuspension in DM . Main cultures were inoculated with washed pre-cultures to an optical density at 595 nm ($OD_{595\ nm}$) of 0.01. Biofilm biomass and chlorophyll fluorescence was determined as described in the main manuscript.

Table S1: Bacterial strains isolated from *in-situ* grown biofilms. Bacterial strains were isolated from PE, PET and PS snippets incubated for five weeks in River *Ems*, Lake *Emssee* and *Rieselfelder*. Strains were assigned to the classes *Alpha*- (■), *Beta*- (■) and *Gammaproteobacteria* (■) or the phyla *Bacteroidetes* (■) and *Actinobacteria* (■) based on the sequence of their 16S rRNA genes. Isolates belonging to the same genus were summarized (*).

Location	Polymer: PE	Polymer: PET	Polymer: PS
Ems	- *Gemmobacter* sp.*<br>- *Hydrogenophaga* sp.*<br>- *Pseudomonas* sp.	- *Gemmobacter* sp.<br>- *Rhizobium* sp.<br>- *Hydrogenophaga* sp.<br>- *Rhodoferax* sp.*<br>- *Flavobacterium* sp.	- *Novosphingobium* sp.*<br>- *Acidovorax* sp.<br>- *Rheinheimera* sp.<br>- *Flavobacterium* sp.
Emssee	- *Gemmobacter* sp.<br>- *Rhodobacter* sp.<br>- *Hydrogenophaga* sp.<br>- *Rhodoferax* sp.<br>- *Pseudomonas* sp.*<br>- *Flavobacterium* sp.	- *Gemmobacter* sp.<br>- *Sphingobium* sp.<br>- *Deefgea* sp.<br>- *Massilia* sp.<br>- *Mitsuaria* sp. (or *Paucibacter* sp.)<br>- *Rhodoferax* sp.<br>- *Pseudomonas* sp.*<br>- *Emticicia* sp.	- *Ciceribacter* sp.<br>- *Gemmobacter* sp.<br>- *Pseudomonas* sp.<br>- *Flavobacterium* sp.*<br>- *Lacibacter* sp.
Rieselfelder	- *Pseudomonas* sp.*<br>- *Chryseobacterium* sp.	- *Gemmobacter* sp. (or *Catellibacterium* sp.)<br>- *Paucibacter* sp. (or *Mitsuaria* sp.)<br>- *Aeromonas* sp.*<br>- *Flavihumibacter* sp.	- *Hydrogenophaga* sp.<br>- *Paucibacter* sp.<br>- *Aeromonas* sp.<br>- *Rheinheimera* sp.<br>- *Plantibacter* sp.

Tab. S2: Bacterial strain collection isolated from photoautotrophic consortia shown in Figure 3 of the main document. Strains were assigned to the classes *Alpha*- (red), *Beta*- (orange) and *Gammaproteobacteria* (yellow) or the phylum *Bacteroidetes* (blue) based on the sequence of their 16S rRNA genes.

Consortium	Strain name	Classification
Co_1	G	(red) *Gemmobacter* sp.
	H, I, J	(orange) *Acidovorax* sp.
Co_2	A	(red) *Gemmobacter* sp.
	B, D	(yellow) *Pseudomonas* sp.
Co_3	W, Z	(red) *Gemmobacter* sp.
	X, Y (2)	(orange) *Acidovorax* sp.
Co_4	S, V	(red) *Rhizobium* sp.
	T (1), T (2), U (1)	(orange) *Hydrogenophaga* sp.
Co_5	M, R	(blue) *Flectobacillus* sp.
	K, L (1), L (2), N (1), N (2), O, P, Q	(red) *Gemmobacter* sp.
Co_6	ZA, ZB, ZD, ZE, ZF	(red) *Gemmobacter* sp.
	ZC	(orange) *Hydrogenophaga* sp.

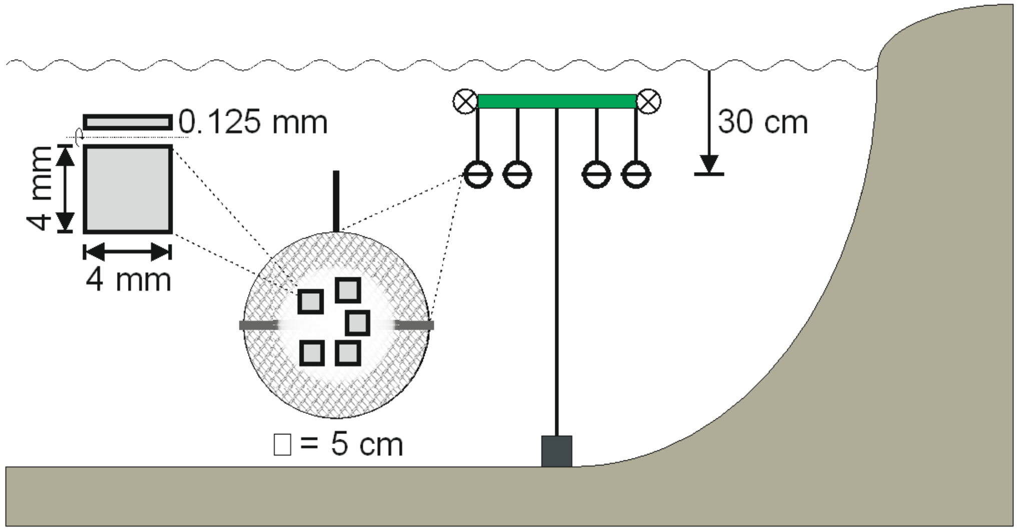

Fig. S1: Schematic overview of *in situ* incubation set-up. 100 plastic snippets (4 × 4 × 0.125 mm) within stainless steel containments (d = 5 cm) were incubated in at a depth of 30 cm for five weeks. For each plastic type, 5 containments were used. Individual containments were removed in weekly intervals for analysis of chlorophyll fluorescence and biofilm biomass (see Fig. 1 in main manuscript). The containments were mounted to foamed polystyrene lifting bodies to a depth of about 30 cm in the respective water columns.

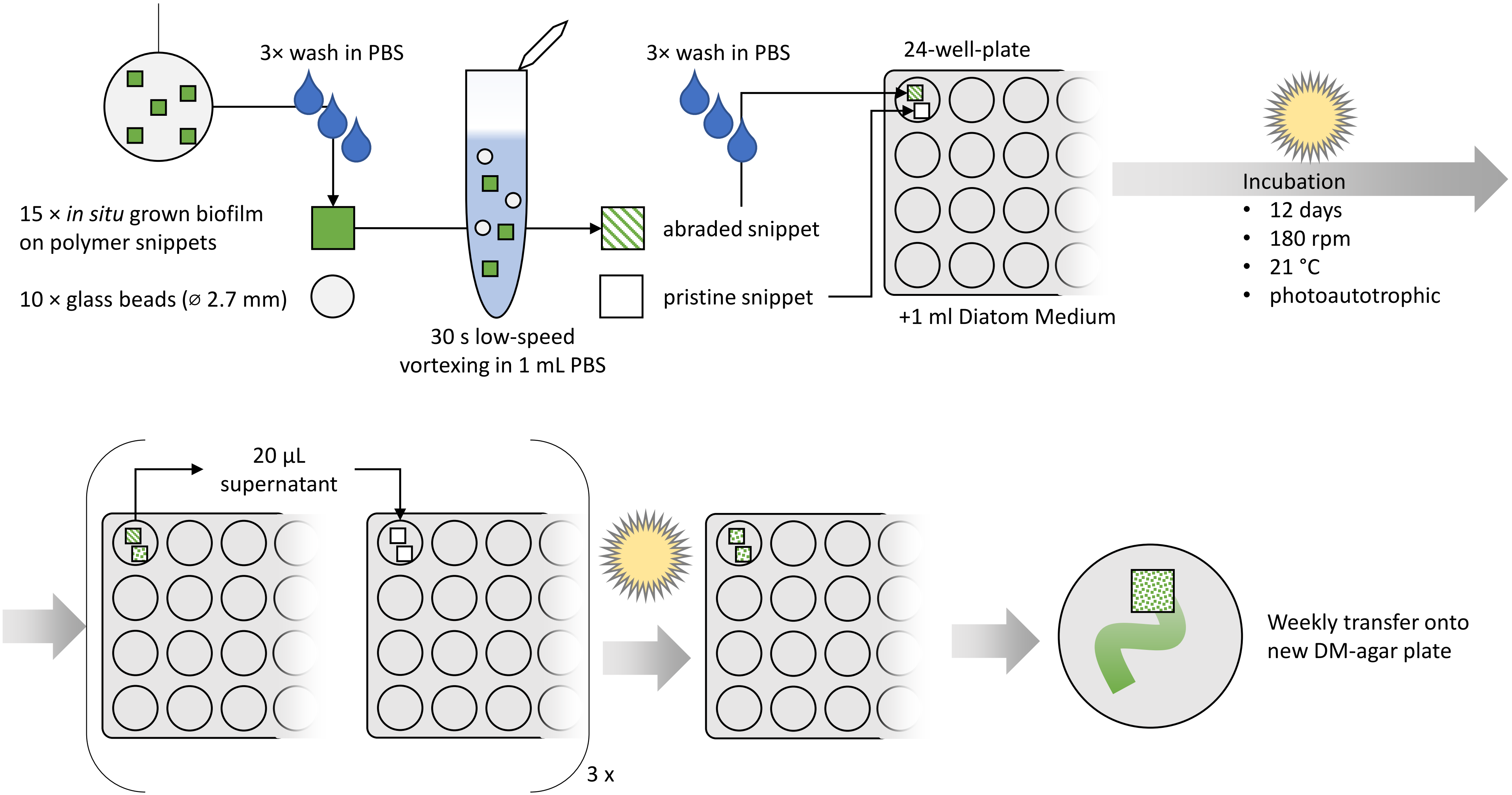

Fig. S2: Flowchart of the enrichment of photoautotrophic consortia on PE-snippets from in-situ grown biofilms. This procedure led to the establishment of consortia Co_1 – Co_6.

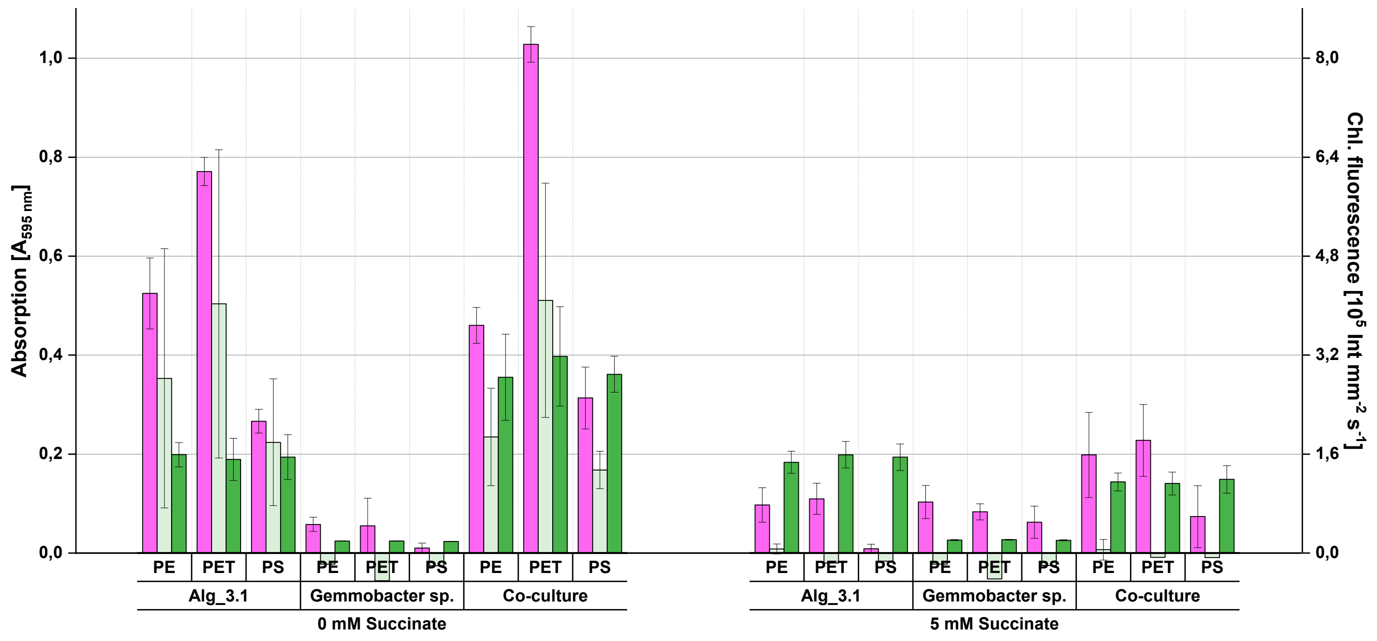

Fig. S3: Biofilm formation by defined dual-species cultures of *Gemmobacter* sp. strain O with strain Alg_3.1 in comparison to the respective mono-cultures: biofilm-biomass (violet) and chlorophyll-fluorescence on plastic snippets (dark-green) and in the culture supernatant (light-green) under photoautotrophic conditions in the absence and presence of succinate as additional carbon and energy source. Error bars indicate standard deviation (n = 8).

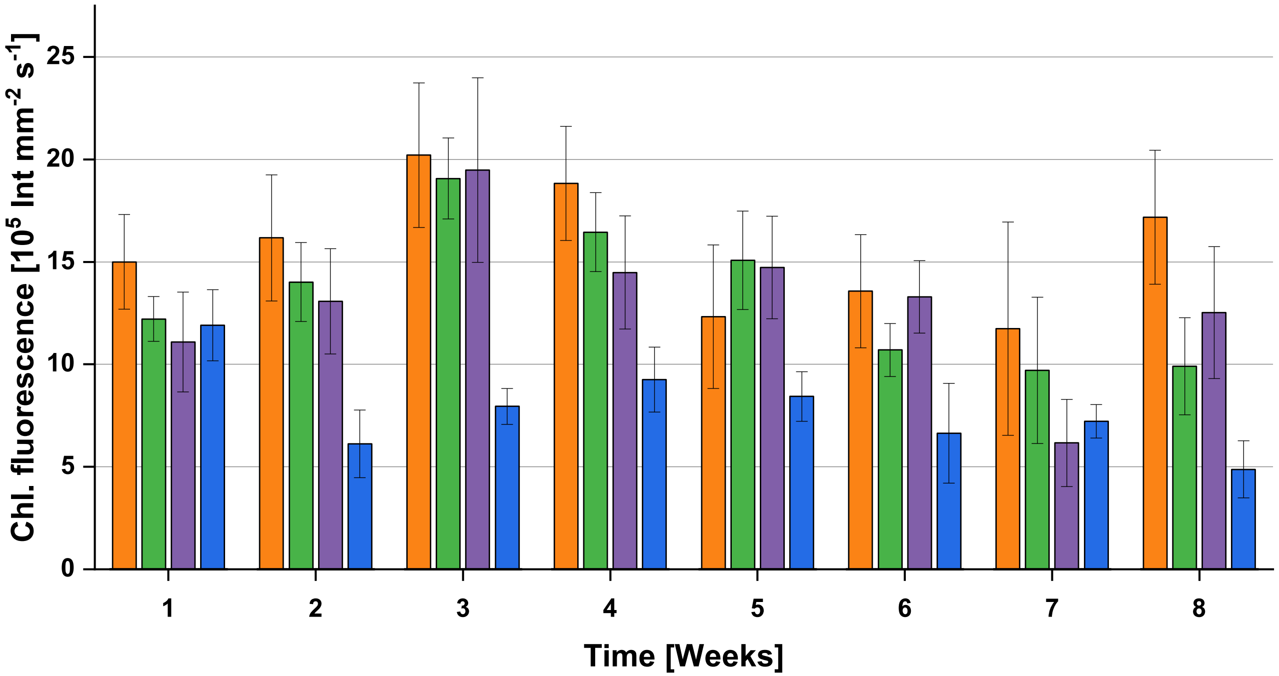

Fig. S4: Chlorophyll fluorescence of biofilms formed during repeated growth experiments of photoautotrophic consortium Co_3 on different materials for usage in grazing experiments. Materials: PE (orange), PET (green), PS (violet) and glass (blue). Error bars indicate standard deviation (n = 16).
